# 643. Evaluation of Rapid Blood Pathogen Identification Along with Antimicrobial Stewardship at an Academic Teaching Institution

**DOI:** 10.1093/ofid/ofab466.840

**Published:** 2021-12-04

**Authors:** Sharon Blum, Terrence McSweeney, Samad Tirmizi, brian Auditore, Diane Johnson, Brian Malone

**Affiliations:** 1 NYU Langone - Long Island, Mineola, NY; 2 Yale New Haven Hospital, New Haven, CT; 4 NYU Langone Long Island, Mineola, New York

## Abstract

**Background:**

Bloodstream infections are a major cause of morbidity and mortality in hospitalized patients. Prompt initiation of effective antimicrobials are essential to optimize patient outcomes. New diagnostic technologies rapidly identifying bacteria, viruses, fungi, and parasites in infections of various body sites. There is a paucity of literature determining if stewardship programs run by one trained pharmacist with rapid diagnostics decreases time to optimal antimicrobial therapy.

**Methods:**

This was a retrospective chart review of positive bloodstream infections identified via rapid diagnostic technologies. The EHR of admitted adult patients with positive BSI identified by BioFire FilmArray Blood Culture Identification (BCID) Panel™ or Accelerate PhenoTest Blood Culture kit™2 between January 2018 – July 2019 were evaluated and pertinent data was collected.

**Results:**

Rapid diagnostic technologies identified 108 bloodstream infections due to gram positive, 56 due to gram negative, and 6 due to *Candida* organisms. Mean time to optimal antimicrobial therapy was significantly lower when pharmacist recommendation was accepted versus when primary care team consulted ID for recommendation or did not accept pharmacist recommendation. Mean time to optimal therapy was 14.7, 34.3, and 271.3 hours (p< 0.0001) respectively. Median total cost of visit per patient, calculated using the average wholesale price of antibiotics multiplied by the number of doses received, was significantly lower when pharmacist recommendations were accepted (&86.40, &147.95, and &239.41, respectively).

Baseline characteristics

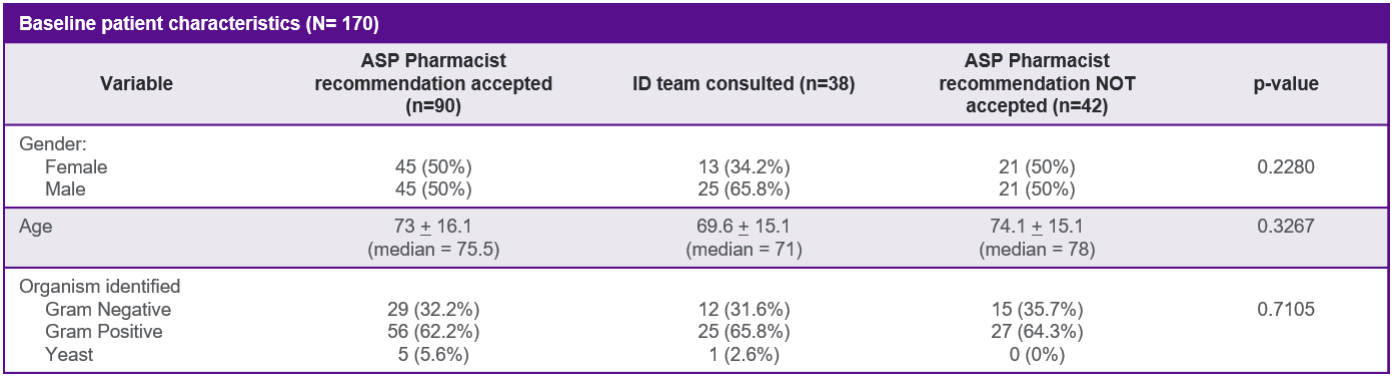

Microbiological isolates

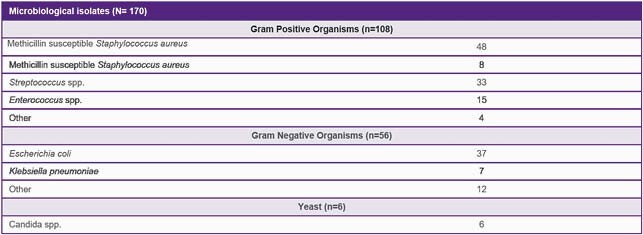

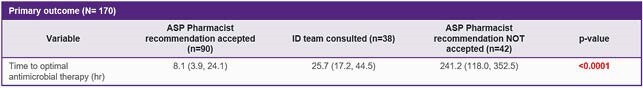

Primary Outcome: Time to Optimal Therapy

**Conclusion:**

The establishment of a pharmacist run antimicrobial stewardship program in conjunction with rapid diagnostic tools for identifying bacteremia led to a decrease in time to optimal antimicrobial therapy and cost savings. Introduction of similar services at community hospitals with limited ASP staffing is justified. Larger studies to further investigate whether ASP partnered with rapid diagnostics have an impact on patient-related outcomes such as mortality and length of stay is warrented.

Secondary outcomes

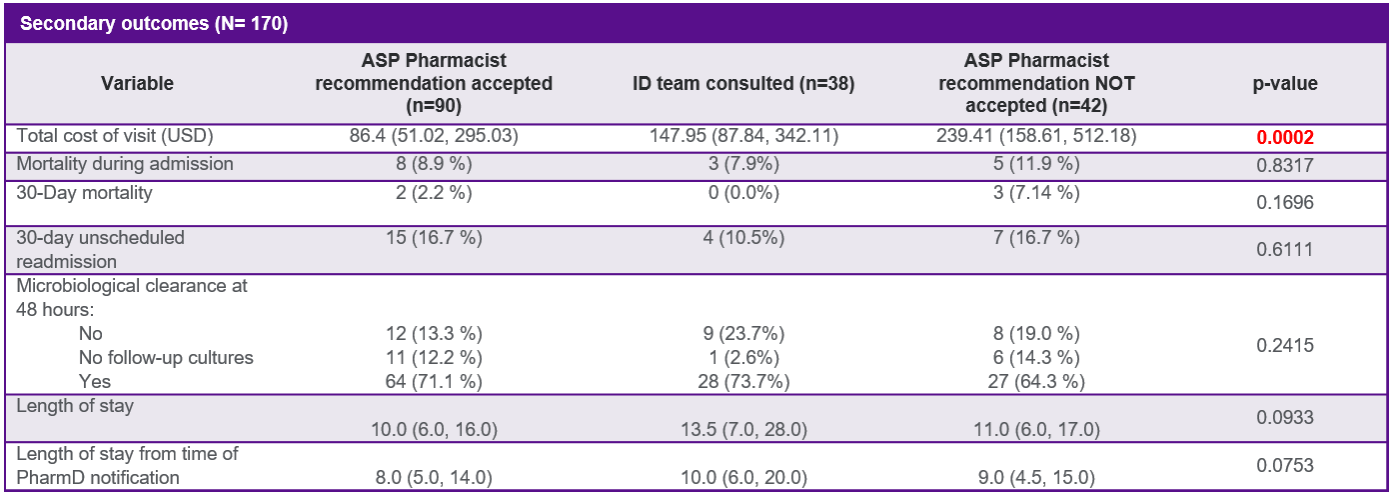

Missed cost savings

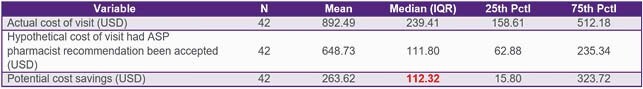

**Disclosures:**

**All Authors**: No reported disclosures

